# Prevalence and Epidemiological Patterns of *Enterobius vermicularis* Infection in Thailand: A Systematic Review and Meta-Analysis

**DOI:** 10.3390/medsci13040207

**Published:** 2025-09-24

**Authors:** Jurairat Jongthawin, Aongart Mahittikorn, Apiporn Thinkhamrop Suwannatrai, Chutima Rattanawan, Kinley Wangdi, Frederick Ramirez Masangkay, Manas Kotepui

**Affiliations:** 1 Faculty of Medicine, Mahasarakham University, Maha Sarakham 44000, Thailand; jurairat.j@msu.ac.th; 2 Department of Protozoology, Faculty of Tropical Medicine, Mahidol University, Bangkok 10400, Thailand; aongart.mah@mahidol.ac.th; 3 Department of Parasitology, Faculty of Medicine, Khon Kaen University, Khon Kaen 40002, Thailand; 4 Department of Medical Science, Amnat Charoen Campus, Mahidol University, Amnat Charoen 37000, Thailand; 5 HEAL Global Research Center, Health Research Institute, Faculty of Health, University of Canberra, Bruce, ACT 2617, Australia; 6 Department of Medical Technology, Faculty of Pharmacy, University of Santo Tomas, Manila 1008, Philippines; frmasangkay@ust.edu.ph; 7 Research Center for the Natural and Applied Sciences, University of Santo Tomas, Manila 1008, Philippines; 8 Medical Technology Program, Faculty of Science, Nakhon Phanom University, Nakhon Phanom 48000, Thailand

**Keywords:** enterobiasis, *Enterobius vermicularis*, Thailand, systematic review, meta-analysis

## Abstract

Background: Enterobiasis, caused by *Enterobius vermicularis*, is recognized as a common intestinal helminthiasis worldwide. Despite multiple surveys in Thailand, no pooled synthesis at the country level has been carried out to evaluate prevalence patterns, temporal trends, or vulnerable groups. Therefore, this systematic review and meta-analysis were undertaken to provide an updated and comprehensive estimate of the prevalence of *E. vermicularis* in Thailand and to identify high-risk populations for targeted interventions. Methods: The systematic review and meta-analysis were conducted in accordance with the Preferred Reporting Items for Systematic Reviews and Meta-Analyses (PRISMA) guidelines (PROSPERO: CRD420251053217). Studies reporting *E. vermicularis* infection in Thailand were systematically searched in international and Thai databases. Pooled prevalence and odds ratios (ORs) were calculated using random-effects models. Subgroup analyses and meta-regression were performed according to year, region, age, population type, and diagnostic method. Results: A total of 56 studies, including 52,765 participants, were analyzed. The overall pooled prevalence was estimated at 3.6% (95% confidence interval [CI]: 2.1–5.9%), with a decline observed in the subgroup analysis by publication year, from 4.75% in 2000–2009 to 1.15% in 2020–2023. The highest prevalence was reported in Central Thailand (7.93%). High infection rates were found among immigrant children (25.2%), hilltribe children (19.9%), Karen students (15.5%), and children in orphanages (11.4%). A markedly higher prevalence was detected by the Scotch tape method compared with direct smear/concentration (12.9% vs. 0.33%). No significant difference in infection risk was observed between males and females (OR = 1.03, *p* = 0.65). Conclusions: The pooled prevalence of *E. vermicularis* in Thailand was estimated at 3.6%, but this figure should be interpreted with caution due to high heterogeneity across studies. More meaningful insights were identified in subgroup analyses, which revealed a temporal decline in prevalence, geographic clustering in Central Thailand, and disproportionately high infection rates among socioeconomically disadvantaged child populations. No statistically significant association was found between gender and risk of infection. These patterns underscore the need for targeted screening, deworming, and hygiene interventions, along with the standardized use of the Scotch tape technique for accurate surveillance and comparability of future studies.

## 1. Introduction

*Enterobiasis* (pinworm infection or oxyuriasis) is one of the most prevalent intestinal helminthiases worldwide and is caused by the nematode *Enterobius vermicularis* [1,2]. The parasite has a simple, direct life cycle, and transmission occurs primarily via the fecal–oral route, autoinfection, or indirect contact with contaminated surfaces or airborne eggs [1,2]. Children are the most affected group due to immature hygiene practices, frequent hand-to-mouth behaviors, and close contact in schools and daycare centers [3]. While most infections are asymptomatic, symptomatic cases may present with perianal pruritus, irritability, abdominal pain, and insomnia [4]. Rarely, ectopic migration can lead to complications such as appendicitis, urinary tract infection, or salpingitis [5,6,7]. Reinfection is common in crowded or resource-limited settings, including orphanages and hilltribe communities, where poor sanitation facilitates ongoing transmission [8].

Globally, *E. vermicularis* remains an underrecognized public health concern, affecting an estimated 200 million children, with over 30% of cases in those aged 5–10 years [9,10]. A recent meta-analysis estimated a pooled global prevalence of 12.9% among children, with the highest rates in Europe (24.9%), followed by South America (14.3%), Asia (13%), Africa (2%), and North America (1.9%) [11]. The same analysis reported substantial heterogeneity, highlighting the need for country-specific epidemiological data to guide targeted control strategies. In Thailand, enterobiasis was historically a major public health issue, with prevalence reaching up to 65% before 2000, particularly in the central region [8,12]. Over the past two decades, improved sanitation, school-based deworming, and health education have contributed to a marked decline in infections [13,14]. Nevertheless, the burden remains disproportionately high among socioeconomically disadvantaged groups, including immigrant children, hilltribe communities, and children in orphanages, reflecting persistent disparities in living conditions, sanitation, and healthcare access [15,16].

Despite several surveys on *E. vermicularis* in Thailand, no comprehensive nationwide synthesis has been conducted to evaluate current prevalence, trends, or residual high-risk populations. This systematic review and meta-analysis aimed to provide an updated estimate of *E. vermicularis* prevalence in Thailand, identify high-risk populations, and highlight gaps for future research.

## 2. Methods

### 2.1. Registration and Protocol

This systematic review and meta-analysis were conducted following the Preferred Reporting Items for Systematic Reviews and Meta-Analyses (PRISMA) guidelines [17]. The protocol was registered in the PROSPERO database (registration number: CRD420251053217).

### 2.2. Search Strategy

A comprehensive search was conducted across six major databases: Journals@Ovid, Nursing & Allied Health Premium, EMBASE, PubMed, Scopus, and Web of Science. The primary search terms included “*Enterobius*” and “Thailand,” and search strategies were tailored slightly to fit each database (see Table S1). To enhance regional coverage, an additional search was performed in the Thai Journal Citation Index (TCI). Furthermore, reference lists of included studies were manually screened for additional relevant articles. The database search was conducted 8–15 May 2025. No restrictions were applied regarding publication date or language.

### 2.3. Eligibility Criteria

Studies were included if they were original research articles that used a cross-sectional study design and reported the prevalence or odds of *E. vermicularis* infection among participants residing in Thailand. Studies were excluded if they were case reports, reviews, books or book chapters, case series, letters, or commentaries; employed cohort or case–control designs; lacked sufficient data for meta-analysis (e.g., missing sample size or number of cases); were published before the year 2000; or were conducted outside Thailand. Studies that reported zero *Enterobius* cases were excluded because most were not specifically designed to detect this parasite and relied on stool-based diagnostic methods with low sensitivity for *E. vermicularis*. Their inclusion could, therefore, lead to an artificial underestimation of prevalence. Additionally, studies were excluded if they focused on treatment or worm retrieval rather than prevalence, or if they used non-standard biological samples for diagnosis (e.g., blood, urine, or serological assays) instead of stool or perianal samples, such as the Scotch tape method.

### 2.4. Study Selection and Data Extraction

All retrieved records were imported into reference management software (EndNote version 21.0, Philadelphia, PA, USA), and duplicate records were removed. Two reviewers (MK and AM) independently screened titles and abstracts against eligibility criteria. Full-text articles were then assessed for inclusion. Disagreements were resolved through discussion. Data extraction was performed by one reviewer (MK) and cross-verified by a second (AM). The following data were extracted: first author, year of publication and data collection, province or region, participant type, age group, gender (if reported), number of participants, number of *E. vermicularis* infections, and diagnostic method used. Data was recorded into a standardized extraction form for consistency.

### 2.5. Risk of Bias Assessment

The risk of bias was assessed using the Joanna Briggs Institute (JBI) critical appraisal checklist for prevalence studies [18]. Two authors (MK and JJ) independently evaluated each study. Risk of bias was categorized as low, moderate, or high based on overall checklist scores, as described previously [19,20]. Discrepancies were resolved through consensus.

### 2.6. Data Synthesis

All quantitative analyses were conducted using RStudio (Version 2024.04.2+764) [21]. A random-effects model (DerSimonian–Laird method) was used to calculate the pooled prevalence and odds ratios (ORs), accounting for between-study variability [22]. Heterogeneity was assessed using the *I*^2^ statistic, with thresholds of 25%, 50%, and 75% indicating low, moderate, and high heterogeneity, respectively [23]. For studies assessing the prevalence of *E. vermicularis*, logit-transformed proportions and standard errors were used in the meta-analysis.

Subgroup analyses were conducted to explore variations by publication year, geographic region, participant age group, population type, and diagnostic method. Meta-regression was performed to assess the influence of these moderators on prevalence estimates. Cumulative meta-analysis was used to examine temporal trends in infection rates. For the association between gender and infection risk, ORs were pooled from 29 studies reporting data stratified by sex. Sensitivity analyses were conducted to assess the robustness of results by applying a fixed-effects model to estimate pooled prevalence and ORs and restricting the analysis to studies that used the Scotch tape technique, considered the most sensitive diagnostic method.

Potential publication bias was evaluated through visual inspection of funnel plots and Egger’s regression test [24]. Funnel plot asymmetry and significant Egger’s test results were considered indicative of publication bias or small-study effects. Analyses were performed separately for the prevalence meta-analysis (all studies), the subset using only the Scotch tape method, and the gender-specific risk analysis.

## 3. Results

### 3.1. Search Results

**Figure 1:**
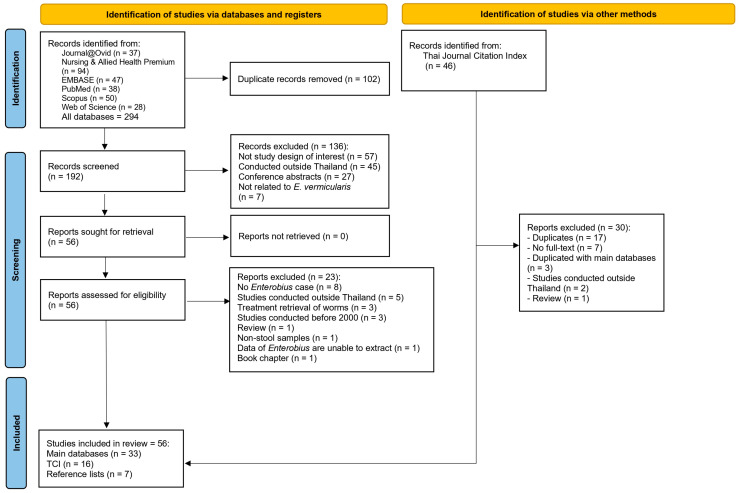
The study flow diagram demonstrates the study selection process.

A total of 294 records were identified from major databases and 46 from the TCI (Figure 1). After removing 102 duplicates, 192 records remained for screening. Of these, 136 were excluded due to reasons such as unsuitable study design, being conducted outside Thailand, or lacking relevance to *E. vermicularis*. The remaining 56 full-text articles were assessed for eligibility, and 23 were excluded due to the absence of *Enterobius* cases, publication year before 2000, or insufficient data for extraction. Finally, 33 eligible studies were identified from the main databases [8,13,15,16,25,26,27,28,29,30,31,32,33,34,35,36,37,38,39,40,41,42,43,44,45,46,47,48,49,50,51,52,53], 16 from TCI [54,55,56,57,58,59,60,61,62,63,64,65,66,67,68,69], and 7 from reference lists [14,70,71,72,73,74,75], resulting in a total of 56 studies included in the systematic review and meta-analysis.

### 3.2. General Characteristics of Included Studies

The 56 studies included in the review were published between 2000 and 2023, with the majority published during 2010–2019 (25/56, 44.6%), followed by 2000–2009 (23/56, 41.1%), and a smaller portion from 2020 to 2023 (8/56, 14.3%). The majority of studies were conducted in Central Thailand (25/56, 44.6%), with fewer from Northeastern (10/56, 17.9%), Northern (8/56, 14.3%), Southern (4/56, 7.14%), and Western Thailand (2/56, 3.57%). School-aged childrenwere the most frequently studied group (34/56, 60.7%), followed by villagers (12/56, 21.4%), and smaller proportions from specific subpopulations such as orphanages, hilltribe children, immigrant children, and Karen students. The majority of studies (42/56, 75.0%) focused on children, while a smaller number involved adults (5/56, 8.93%) or both children and adults (9/56, 16.1%). The Scotch tape technique was the most frequently used method for detecting *E. vermicularis* 35/56, 62.5%), while the direct smear or concentration technique was used for the rest of the studies (21/56, 37.5%, Table 1).

### 3.3. Risk of Bias Assessment Results

The majority of studies showed a low risk of bias (37/56, 66.1%), while the remaining studies had a moderate risk of bias (19/56, 33.9%; Table S3). No study was classified as having a high risk of bias.

### 3.4. Prevalence Estimate of E. vermicularis Infections

**Figure 2:**
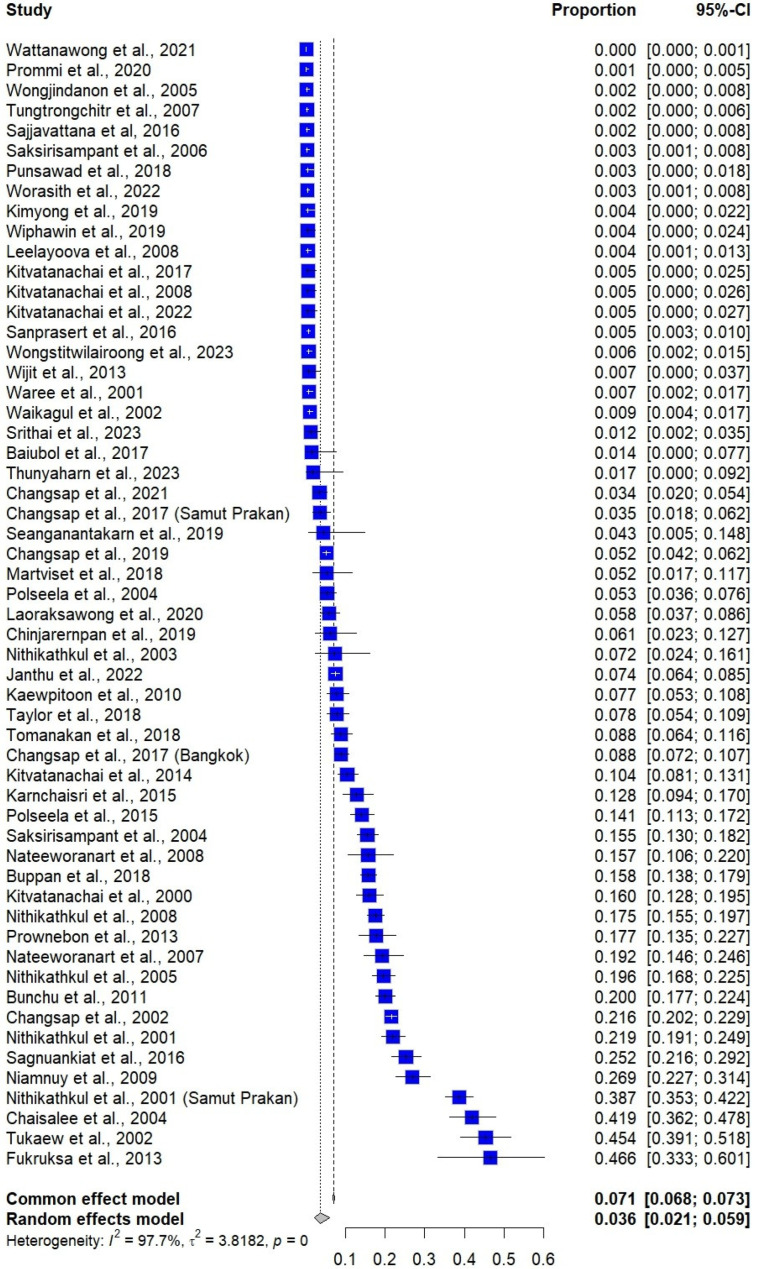
Forest plot showing the prevalence of *E. vermicularis* infections among Thai participants from 56 studies. Each horizontal line represents a 95% confidence interval (CI) for the prevalence reported in an individual study, with blue squares indicating the point estimates and their relative weights in the meta-analysis. The pooled prevalence was estimated using both common (fixed) and random-effects models [8,13,14,15,16,25,26,27,28,29,30,31,32,33,34,35,36,37,38,39,40,41,42,43,44,45,46,47,48,49,50,51,52,53,54,55,56,57,58,59,60,61,62,63,64,65,66,67,68,69,70,71,72,73,74,75].

The pooled prevalence estimate of *E. vermicularis* infections among Thai participants (52,765 participants) using a random-effects model was 3.6% (95% confidence interval [CI]: 2.10–5.90%, 56 studies, 3724 infected cases). The high heterogeneity (*I*^2^ = 97.7%) suggests considerable differences among studies (Figure 2).

**Figure 3:**
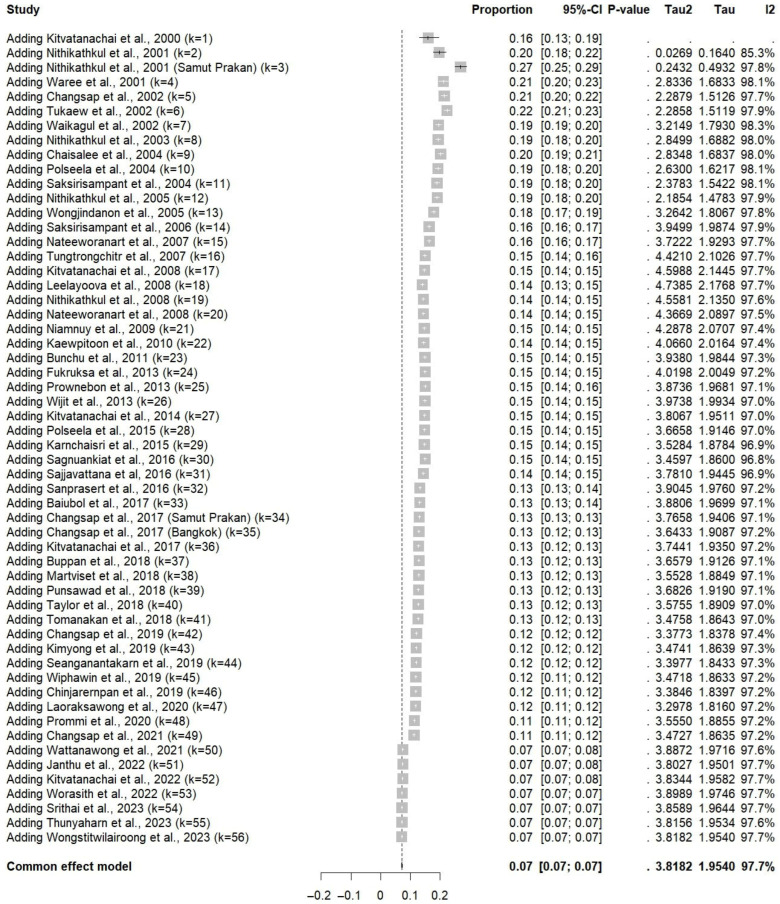
Cumulative meta-analysis of the prevalence of *E. vermicularis* infections among Thai participants over time. Studies are added sequentially by publication year to assess temporal trends in pooled prevalence estimates. Each row represents the updated pooled proportion and 95% confidence interval (CI) after including the corresponding study [8,13,14,15,16,25,26,27,28,29,30,31,32,33,34,35,36,37,38,39,40,41,42,43,44,45,46,47,48,49,50,51,52,53,54,55,56,57,58,59,60,61,62,63,64,65,66,67,68,69,70,71,72,73,74,75].

The cumulative meta-analysis of *E. vermicularis* infections in Thai participants indicates a declining trend in prevalence over time. Early studies from the 2000s reported relatively high estimates, but as more recent studies are added, the cumulative prevalence gradually decreases and stabilizes around 7% (Figure 3). Additionally, the meta-regression analysis identified publication year as a significant moderator (*p* = 0.0015), indicating a temporal decline in the reported prevalence of *E. vermicularis* infections in Thailand. Specifically, more recent studies tend to report lower prevalence rates (Figure 3).

**Figure 4:**
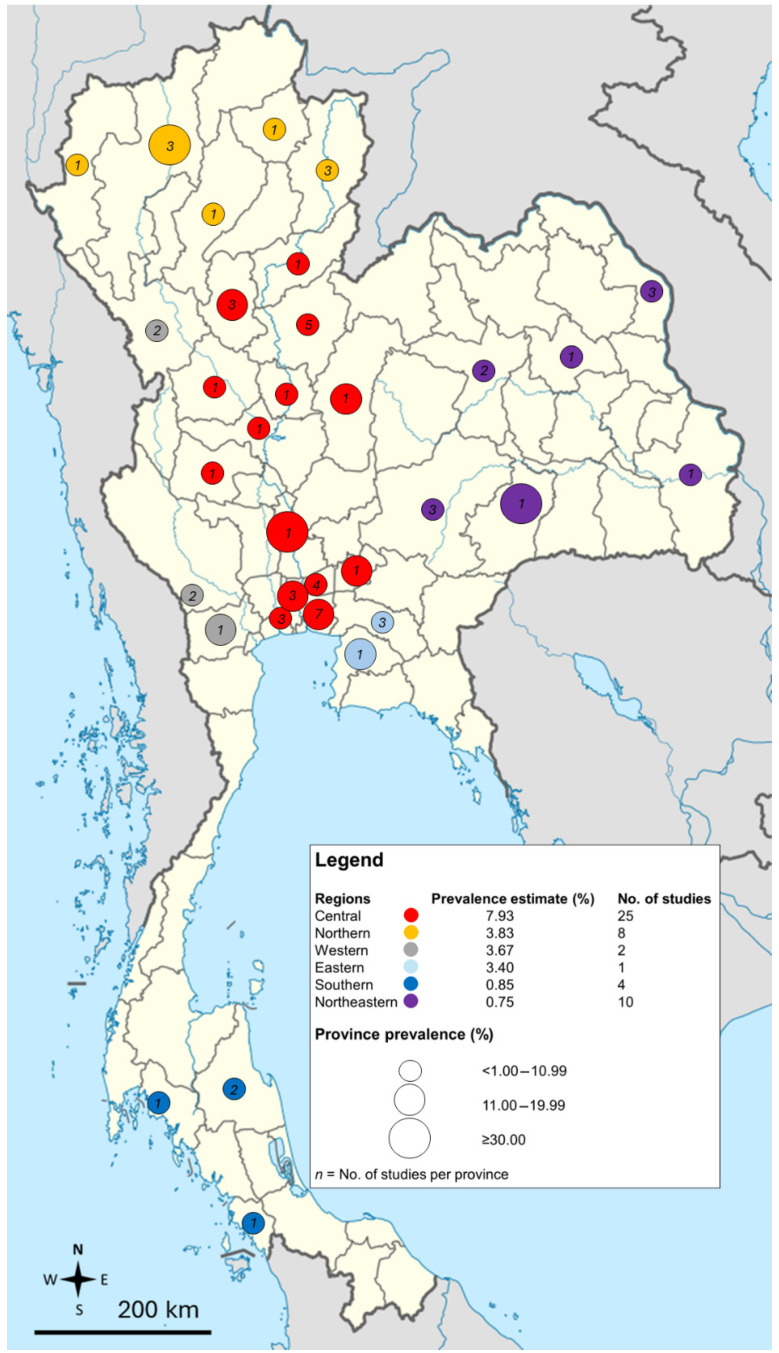
Geographic distribution of the prevalence estimates of *E. vermicularis* infections in Thailand. Data from multi-province studies are excluded from the map. Map of Thailand (Thailand location map.svg) was sourced license-free from Wikimedia Commons: https://commons.wikimedia.org/w/index.php?search=thailand+map&title=Special:MediaSearch&go=Go&type=image (accessed on 12 August 2025) and annotated by the authors.

The meta-regression and subgroup analyses revealed several key factors contributing to the variation in the reported prevalence of *E. vermicularis* infections in Thailand. Meta-regression identified publication year, geographic region, age group, participant type, and detection method as significant moderators (*p* < 0.05, Table S4), indicating that these variables significantly influenced prevalence estimates, while the proportion of male participants showed no significant effect (*p* = 0.16). Subgroup analysis showed that the pooled prevalence declined over time, with the highest rates observed between 2000 and 2009 (4.75%) and the lowest from 2020 to 2023 (1.15%) (Table S4). Subgroup analysis, including regions with more than one study, showed that Central Thailand had the highest prevalence (7.93%), followed by Northern Thailand (3.83%), Western Thailand (3.67%), Southern Thailand (0.85%), and Northeastern Thailand (0.75%). Prevalences and distributions of *E. vermicularis* infections in several provinces of Thailand are demonstrated in Figure 4 (Table S4).

Children were significantly more affected than adults (7.05% vs. 0.45%), and the highest infection rates were observed among vulnerable groups such as immigrant children (25.2%), hilltribe children (19.9%), Karen students (15.5%, referring to children of the Karen ethnic minority group in Northern and Western Thailand), and children in orphanages (11.4%). Furthermore, studies using the Scotch tape technique reported a much higher prevalence than those using direct smear or concentration methods (12.9% vs. 0.33%), suggesting the impact of diagnostic sensitivity.

### 3.5. Gender and Risk of E. vermicularis Infections

**Figure 5:**
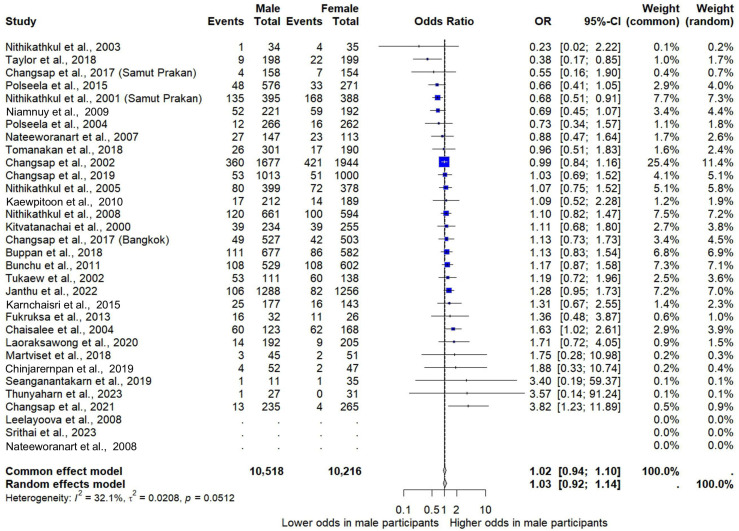
Pooled odds ratio (OR) comparing the risk of *E. vermicularis* infection between male and female participants in Thailand. Each study is represented with its OR and 95% confidence interval (CI), comparing the odds of infection in males versus females. An OR > 1 indicates higher odds in males, and an OR < 1 indicates higher odds in females. The summary estimates are shown at the bottom using both common (fixed)-effects and random-effects models [8,14,16,25,26,27,31,32,33,35,36,37,38,44,45,55,56,57,58,61,63,64,66,67,68,69,70,71,72,73,74,75].

Based on the results from 29 studies with a combined sample size of 20,734 individuals, the association between sex and the risk of *E. vermicularis* infection in Thailand was assessed. Results showed no statistically significant difference in infection risk between males and females (*p* = 0.65, OR: 1.03, 95% CI: 0.92–1.14, *I*^2^: 32.1%, 29 studies, Figure 5). The meta-regression and subgroup analyses further suggested that none of the covariates significantly affected the pooled OR (*p* > 0.05 in all subgroup analyses, Table S5).

### 3.6. Sensitivity Analysis

The sensitivity analysis using the fixed-effects model showed that the pooled prevalence estimate of *E. vermicularis* infections among Thai participants is 7.06 (95% CI: 6.84–7.28, 56 studies, Figure 2). In addition, the sensitivity analysis excluding studies that did not use the Scotch tape technique for the diagnostic method showed that the pooled prevalence estimate of *E. vermicularis* infections among Thai participants is 12.9% (95% CI: 9.90–16.59, *I*^2^: 97.4%, 35 studies, Table S4). For the association between gender and the odds of *E. vermicularis* infection in Thailand, the fixed-effects models also showed a similar non-significant result (*p* = 0.64; OR = 1.02, 95% CI: 0.94–1.10; Figure 5).

### 3.7. Publication Bias

**Figure 6:**
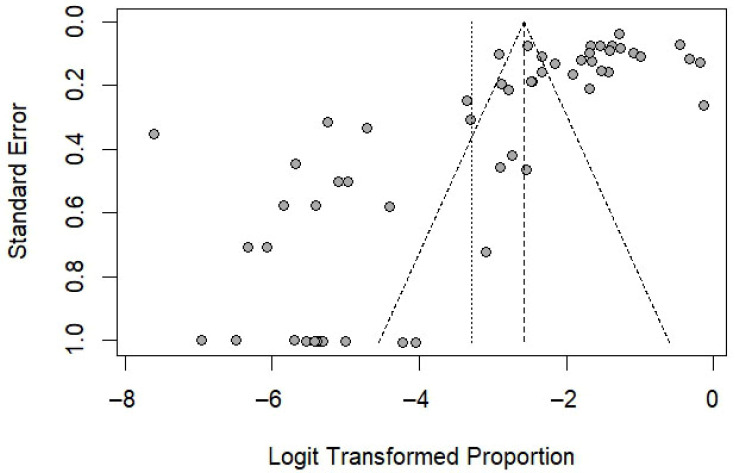
Funnel plot of logit-transformed proportions against standard errors for studies included in the meta-analysis (56 studies). Cycles represent individual studies. The vertical dashed line indicates the pooled estimate, while the diagonal dashed lines represent the pseudo 95% confidence limits. The plot shows asymmetry, indicating potential publication bias or small-study effects in the meta-analysis.

**Figure 7:**
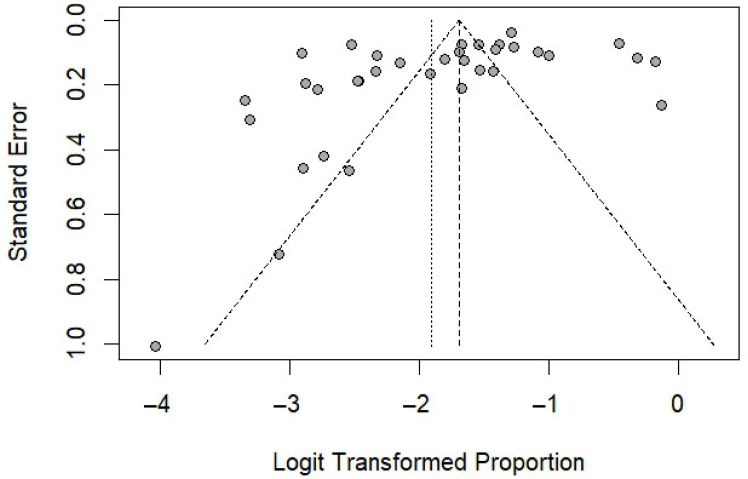
Funnel plot of logit-transformed proportions against standard errors for studies included in the meta-analysis (35 studies). Cycles represent individual studies. The vertical dashed line indicates the pooled estimate, while the diagonal dashed lines represent the pseudo 95% confidence limits.

**Figure 8:**
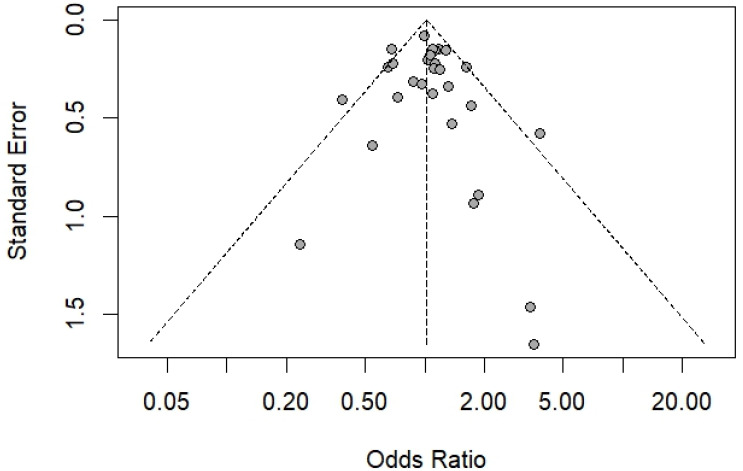
Funnel plot of logit-transformed proportions against standard errors for studies included in the meta-analysis (29 studies). Cycles represent individual studies. The vertical dashed line indicates the pooled estimate, while the diagonal dashed lines represent the pseudo 95% confidence limits.

For the pooled prevalence analysis, the funnel plot demonstrated asymmetry (Figure 6), and Egger’s test (t = –5.68, *p* < 0.0001) provided strong statistical evidence of this bias. These results indicate potential publication bias or small-study effects in the meta-analysis. When the analysis was limited to studies that used the Scotch tape technique as the diagnostic method, the funnel plot still showed asymmetry (Figure 7), and Egger’s test remained statistically significant (*p* = 0.038), further supporting the presence of potential publication bias or small-study effects. For the association between sex and the risk of *E. vermicularis* infection in Thailand, the funnel plot demonstrated symmetry (Figure 8), with Egger’s test revealing no statistically significant result (*p* = 0.53), suggesting the absence of potential publication bias or small-study effects.

## 4. Discussion

This systematic review and meta-analysis, which included 56 studies with over 52,000 participants, estimated the pooled prevalence of *E. vermicularis* infection in Thailand at 3.6%. This prevalence is lower than the global average of 12.9%, as reported in a previous meta-analysis [11]. In that earlier meta-analysis, only two studies from Thailand investigating *E. vermicularis* infection in young children were included. In contrast, the present study included a much larger number of studies, further supporting that the prevalence in Thailand is relatively low compared to both the Asian regional estimate (13%) and the global prevalence (12.9%) [11]. Nevertheless, the high heterogeneity across studies (*I*^2^ = 97.7%) suggested that local context, population characteristics, and methodological variations may influence reported *E. vermicularis* infection prevalences. In neighboring countries such as the Lao People’s Democratic Republic (PDR) and Cambodia, the reported prevalence of *E. vermicularis* infection is generally low, likely due to the use of less sensitive diagnostic methods such as the Kato–Katz thick smear. For example, a study of villagers in Lao PDR reported a low prevalence of 1% using this technique [76]. Similarly, a survey among children under five years of age in Lao PDR found a prevalence of just 1% [77]. In Cambodia, the prevalence among villagers was also low (1.0%) when using the Kato–Katz method [78]. In contrast, a study from Myanmar that employed the more sensitive Scotch tape technique reported a much higher prevalence of 47.2% among schoolchildren [79].

The majority of studies on *E. vermicularis* originated from Central Thailand (44.6%), particularly Bangkok and its surrounding metropolitan areas. This concentration likely reflects the region’s well-established research infrastructure and access to advanced parasitology laboratories. Moreover, the high urban population in Bangkok and adjacent provinces, such as Pathum Thani [80,81], is associated with large schools that have high student densities, potentially increasing the risk of infection and providing researchers with easier access to study participants. In addition, the availability of funding from institutions that support research in this region may have further contributed to the focus on Central Thailand. By contrast, studies from more rural or remote regions, such as Northern, Northeastern, and Southern Thailand, were relatively few. This likely reflects limited research resources, logistical challenges, and smaller population centers, which may reduce the feasibility of conducting large-scale surveys. Consequently, while Central Thailand contributed the largest body of evidence, these geographic imbalances should be considered when interpreting the national prevalence estimates.

The systematic review and meta-analysis revealed that over 60% of the study targets were school-aged children, who are at greatest risk for *E. vermicularis* infection due to behaviors such as nail-biting, finger-sucking, and poor hand hygiene [82]. Although the majority of studies enrolled school-aged children, the pooled prevalence of *E. vermicularis* infection in this group was 5.03%. This prevalence was lower than that observed in other child populations, such as children in communities (20.78%), hilltribe children (19.93%), those in orphanages (11.4%), hilltribe children in orphanages (17.73%), immigrant children (25.24%), and Karen students (15.49%). These findings suggest that children from specific vulnerable or marginalized populations in Thailand, often facing social, economic, or geographic disadvantages, are at a substantially higher risk of *E. vermicularis* infection.

The cumulative meta-analysis and meta-regression identified a significant temporal decline in the prevalence of *E. vermicularis*, with more recent studies reporting lower prevalences. This trend may reflect improvements in healthcare access, sanitation, and awareness, particularly among school-aged children who comprised the majority of the study populations. Nevertheless, despite this decline, *E. vermicularis* infection remains a persistent concern in certain subgroups. Subgroup analyses revealed higher prevalence among immigrant children, hilltribe communities, Karen students, and children in orphanages. The persistently high prevalence of *E. vermicularis* infection in these groups is likely due to socioeconomic disadvantages, inadequate sanitation, household overcrowding, limited access to healthcare and deworming programs, and cultural and language barriers that hinder effective hygiene education. An intervention study demonstrated that home visits combined with health education for mothers and children significantly improved knowledge, enhanced hygiene practices, and reduced parasite burden [83]. Such health education programs are particularly needed for high-risk groups, including hilltribe communities, immigrant children, Karen students, and children in orphanages. Evidence from a previous interventional study showed that children who received education on *E. vermicularis* transmission and preventive practices had a lower prevalence of infection compared to those who did not receive any educational intervention [84].

Geographically, this systematic review and meta-analysis found that most studies were conducted in Central Thailand, which also reported the highest prevalence of *E. vermicularis* infection (7.93%), followed by the Northern (3.83%) and Western (3.67%) regions. In contrast, the Northeastern (0.75%) and Southern (0.85%) regions showed relatively low prevalence rates. These regional differences may reflect variations in living conditions, population density, and healthcare infrastructure. For example, Central Thailand has a higher population density and more crowded school settings, which can facilitate the transmission of the parasite. Meanwhile, the lower prevalence in the Northeastern and Southern regions may be due to less dense populations, better hygiene practices, or the use of less sensitive diagnostic methods, such as not employing the Scotch tape technique. Additionally, under-reporting in regions with limited surveillance capacity could contribute to the observed disparities.

The detection method significantly affected prevalence estimates. Studies using the Scotch tape technique reported markedly higher prevalence than those relying on direct smear or concentration techniques (12.88% vs. 0.33%), suggesting that studies not employing the Scotch tape method may underestimate the true prevalence of *E. vermicularis* infections. *E*. *vermicularis* infections may incidentally be detected by methods of parasitological surveys, such as direct smears or concentration techniques, as demonstrated by previous studies [28,29,54,59]. However, the Scotch tape technique is regarded as the gold standard for detecting *E. vermicularis* because the eggs are deposited on the perianal skin rather than being commonly found in stool samples [1]. Its low cost, simplicity, and high sensitivity make it particularly well-suited for use in school and community-based surveillance programs. In interpreting the observed decline in prevalence over time, it should be noted that diagnostic heterogeneity may have influenced these estimates. Earlier studies often relied on less sensitive stool-based methods, which likely underestimated the true prevalence of *E. vermicularis* infections. In contrast, studies employing the Scotch tape technique, recognized as the gold standard, reported substantially higher prevalence rates. This difference in sensitivity creates a risk of underestimation when stool-based methods are used, particularly in large-scale parasitological surveys where pinworm detection was not the primary objective. Conversely, the apparent decline in prevalence over time could partly reflect a shift in study designs and populations investigated, rather than a true reduction in transmission. Therefore, while the overall trend suggests a declining prevalence in Thailand, caution is warranted when interpreting temporal patterns, as changes in diagnostic methods may have contributed to the observed evolution.

Regarding gender, the analysis found no statistically significant difference in the risk of *E. vermicularis* infection between males and females. This result remained consistent across sensitivity and subgroup analyses, suggesting that gender is unlikely to be a major determinant of *E. vermicularis* infection in the Thai context, despite some previous studies reporting higher infection rates among males [64,70,74]. The overall meta-analysis indicates that *E. vermicularis* infection is independent of gender, possibly due to shared group activities among children, such as playing on the floor, communal eating habits, or taking naps together, which facilitate equal exposure regardless of sex.

The findings of this study have important public health and research implications. First, while the overall prevalence of *E. vermicularis* infection in Thailand appears to be lower than global and regional estimates, the infection remains a significant concern among high-risk groups such as children, particularly those in orphanages, hill tribes, and migrant communities. Second, the study highlights the critical role of diagnostic methods in prevalence estimation. The significantly higher prevalence observed in studies using the Scotch tape technique suggests that reliance on less sensitive methods may lead to underestimation of infection burden. Third, the geographic differences in prevalence point to the need for region-specific public health strategies, especially in Central Thailand, where prevalence rates are highest. Public health efforts should prioritize targeted screening, treatment, and hygiene promotion in these groups. The adoption of standardized diagnostic methods, particularly the Scotch tape technique, is essential for accurate surveillance and effective control of *E. vermicularis* infections in epidemiological settings. However, the number of intervention evaluation studies in Thailand remains limited, restricting conclusions about the most effective public health measures. Further interventional research, including large-scale deworming programs and community-based hygiene interventions, is required to guide evidence-based control strategies.

This systematic review and meta-analysis had several limitations. First, substantial heterogeneity was observed in the prevalence estimates, which may reflect regional differences. Therefore, the relative patterns across subgroups—such as time, region, population type, and diagnostic method—likely provide the most reliable epidemiological insights and should be prioritized when interpreting the findings. Second, evidence of publication bias was detected in the prevalence analysis, suggesting that studies reporting very low or non-significant prevalence were less likely to be published. Consequently, the true national prevalence of enterobiasis may be lower than the pooled estimate. However, no significant bias was found in the gender-based comparison. Third, the prevalence in some regions may have been underestimated or overestimated due to the small number of available studies. Importantly, the geographic distribution of studies was uneven, with most conducted in Central Thailand, where higher population density and greater research infrastructure facilitated large-scale investigations. These studies also more frequently employed the Scotch tape technique, a more sensitive diagnostic method than stool-based smear or concentration techniques commonly used in rural or remote areas. This combination of geographic imbalance and diagnostic heterogeneity introduces an important bias that may exaggerate apparent regional differences. Fourth, one important limitation of this study is the exclusion of research reporting zero cases of enterobiasis. This decision was primarily based on methodological considerations: many of these studies employed diagnostic methods with low sensitivity, such as stool examination, and were not specifically designed to detect *E. vermicularis.* While this approach ensured methodological consistency and reliability in the included studies, it may have introduced selection bias, potentially leading to an overestimation of the pooled prevalence. Therefore, the true national prevalence of enterobiasis could be lower than the estimate.

## 5. Conclusions

The pooled prevalence of *E. vermicularis* in Thailand was estimated at 3.6%, but this figure should be interpreted with caution, given the high heterogeneity across studies. More meaningful insights were found in subgroup analyses, which showed a clear temporal decline in prevalence, geographic clustering in Central Thailand, and disproportionately high infection rates among socioeconomically disadvantaged child populations. No statistically significant association was found between gender and risk of infection. These epidemiological patterns highlight the need for targeted screening, deworming, and hygiene interventions, as well as the standardized use of the Scotch tape technique to ensure accurate surveillance and comparability of future studies.

## Figures and Tables

**Table 1:** Summary characteristics of the 56 studies included in the systematic review.

Publication Year	*n*	%
- 2000–2009	23	41.1
- 2010–2019	25	44.6
- 2020–2023	8	14.3
**Parts of Thailand**		
- Central Thailand	25	44.6
- Northeastern Thailand	10	17.9
- Northern Thailand	8	14.3
- Southern Thailand	4	7.14
- Western Thailand	2	3.57
- Central and Western Thailand	1	1.79
- Central, Northeastern Thailand	1	1.79
- Central, Northeastern, Northern, Eastern, Western, and Southern Thailand	1	1.79
- Central, Western, and Eastern Thailand	1	1.79
- Central, Northeastern, Northern, Eastern, and Western Thailand	1	1.79
- Eastern Thailand	1	1.79
- All areas	1	1.79
**Participants**		
- School children	34	60.7
- Villagers	12	21.4
- Orphanages	2	3.57
- Hilltribe children	2	3.57
- Children in communities	2	3.57
- Hilltribe children/Orphanage	1	1.79
- School-aged children/Orphanages	1	1.79
- Immigrant children	1	1.79
- Karen students	1	1.79
**Age groups**		
- Children	42	75.0
- Adults	5	8.93
- Children and adults	9	16.1
**Detection method**		
- Scotch tape technique	35	62.5
- Direct smear/Concentration technique	21	37.5

## Data Availability

All data generated or analyzed during this study are included in this manuscript and its Supplementary Tables S1–S5.

## Supplementary Materials

The following supporting information can be downloaded at: https://www.mdpi.com/article/10.3390/medsci13040207/s1, Supplementary Tables: Table S1. Search terms. Table S2. Details of included studies. Table S3. Risk of bias. Table S4. Meta-regression and subgroup analysis of the pooled prevalence of *E. vermicularis* infections in Thailand. Table S5. Meta-regression and subgroup analysis of the pooled odds ratio (OR) comparing the risk of *E. vermicularis* infections between male and female participants in Thailand.
