# Supplementary material for: Prevalence and Epidemiological Patterns of Enterobius vermicularis Infection in Thailand: A Systematic Review and Meta-Analysis
Source: Med Sci (Basel). 2025 Sep 24;13(4):207. doi: 10.3390/medsci13040207 (PMC12551123; doi:10.3390/medsci13040207)
Supplement: Supplementary file 1 [file medsci-13-00207-s001.zip › Table S1. Search terms.pdf]

Table S1. Search terms

General keywords

(Enterobius OR Pinworms OR Pinworm OR Threadworms OR Threadworm OR “Enterobius vermicularis” OR “Enterobius vermiculari” OR “Oxyuris vermicularis” OR “Oxyuris vermiculari”) AND (Thailand OR Siam)

PubMed 8 May 2025

No.	Key concept	Search terms	Results
1.	*Enterobius*	Enterobius[MeSH Terms] OR Pinworms[MeSH Terms] OR Pinworm[MeSH Terms] OR Threadworms[MeSH Terms] OR Threadworm[MeSH Terms] OR “Enterobius vermicularis”[MeSH Terms] OR “Enterobius vermiculari”[MeSH Terms] OR “Oxyuris vermicularis”[MeSH Terms] OR “Oxyuris vermiculari”[MeSH Terms] OR Enterobius[Text Word] OR Pinworms[Text Word] OR Pinworm[Text Word] OR Threadworms[Text Word] OR Threadworm[Text Word] OR “Enterobius vermicularis”[Text Word] OR “Enterobius vermiculari”[Text Word] OR “Oxyuris vermicularis”[Text Word] OR “Oxyuris vermiculari”[Text Word]	2,626
2.	Thailand	Thailand[Text Word] OR Thailand[MeSH Terms] OR Siam[Text Word] OR Siam[MeSH Terms]	49,092
3.	#1 AND #2	(Enterobius[MeSH Terms] OR Pinworms[MeSH Terms] OR Pinworm[MeSH Terms] OR Threadworms[MeSH Terms] OR Threadworm[MeSH Terms] OR “Enterobius vermicularis”[MeSH Terms] OR “Enterobius vermiculari”[MeSH Terms] OR “Oxyuris vermicularis”[MeSH Terms] OR “Oxyuris vermiculari”[MeSH Terms] OR Enterobius[Text Word] OR Pinworms[Text Word] OR Pinworm[Text Word] OR Threadworms[Text Word] OR Threadworm[Text Word] OR “Enterobius vermicularis”[Text Word] OR “Enterobius vermiculari”[Text Word] OR “Oxyuris vermicularis”[Text Word] OR “Oxyuris vermiculari”[Text Word]) AND (Thailand[Text Word] OR Thailand[MeSH Terms] OR Siam[Text Word] OR Siam[MeSH Terms])	38

Embase 8 May 2025

No.	Key concept	Search terms	Results
1.	*Enterobius*	'enterobius'/exp OR pinworms OR 'pinworm'/exp OR threadworms OR 'threadworm'/exp OR 'enterobius vermicularis'/exp OR 'enterobius vermiculari' OR 'oxyuris vermicularis'/exp OR 'oxyuris vermiculari' OR enterobius:ti,ab,kw,de OR pinworms:ti,ab,kw,de OR pinworm:ti,ab,kw,de OR threadworms:ti,ab,kw,de OR threadworm:ti,ab,kw,de OR 'enterobius vermicularis':ti,ab,kw,de OR 'enterobius vermiculari':ti,ab,kw,de OR 'oxyuris vermicularis':ti,ab,kw,de OR 'oxyuris vermiculari':ti,ab,kw,de	3573
2.	Thailand	thailand:ti,ab,kw,de OR 'thailand'/exp OR siam:ti,ab,kw,de OR 'siam'/exp	57403
3.	#1 AND #2	('enterobius'/exp OR pinworms OR 'pinworm'/exp OR threadworms OR 'threadworm'/exp OR 'enterobius vermicularis'/exp OR 'enterobius vermiculari' OR 'oxyuris vermicularis'/exp OR 'oxyuris vermiculari' OR enterobius:ti,ab,kw,de OR pinworms:ti,ab,kw,de OR pinworm:ti,ab,kw,de OR threadworms:ti,ab,kw,de OR threadworm:ti,ab,kw,de OR 'enterobius vermicularis':ti,ab,kw,de OR 'enterobius vermiculari':ti,ab,kw,de OR 'oxyuris vermicularis':ti,ab,kw,de OR 'oxyuris vermiculari':ti,ab,kw,de) AND (thailand:ti,ab,kw,de OR 'thailand'/exp OR siam:ti,ab,kw,de OR 'siam'/exp)	47

Scopus 15 May 2025

No.	Key concept	Search terms	Results
1.	*Enterobius*	TITLE-ABS-KEY (enterobius OR pinwormsOR pinworm OR threadworms OR threadworm OR "Enterobius vermicularis" OR "Enterobius vermiculari" OR "Oxyuris vermicularis" OR "Oxyuris vermiculari")	4,047
2.	Thailand	TITLE-ABS-KEY (thailand OR siam)	133,557
3.	1 AND 2	(TITLE-ABS-KEY (enterobius OR pinworms OR pinworm OR threadworms OR threadworm OR "Enterobius vermicularis" OR "Enterobius vermiculari" OR "Oxyuris vermicularis" OR "Oxyuris vermiculari")) AND (TITLE-ABS-KEY (thailand OR siam))	50

Ovid (Journal) 8 May 2025

No.	Key concept	Search terms	Results

1.	*Enterobius* AND Thailand	(Enterobius OR Pinworms OR Pinworm OR Threadworms OR Threadworm OR "Enterobius vermicularis" OR "Enterobius vermiculari" OR "Oxyuris vermicularis" OR "Oxyuris vermiculari") AND (Thailand OR Siam) {Including Limited Related Terms}	37

Nursing & Allied Health Premium 8 May 2025

No.	Key concept	Search terms	Results
1.	*Enterobius* AND Thailand	(Enterobius OR Pinworms OR Pinworm OR Threadworms OR Threadworm OR “Enterobius vermicularis” OR “Enterobius vermiculari” OR “Oxyuris vermicularis” OR “Oxyuris vermiculari”) AND (Thailand OR Siam)	94

Web of Science 8 May 2025

No.	Key concept	Search terms	Results
1.	*Enterobius* AND Thailand	(Enterobius OR Pinworms OR Pinworm OR Threadworms OR Threadworm OR “Enterobius vermicularis” OR “Enterobius vermiculari” OR “Oxyuris vermicularis” OR “Oxyuris vermiculari”) AND (Thailand OR Siam)	28

Thai-Journal Citation Index 8 May 2025

No.	Key concept	Search terms	Results
1.	*Enterobius*	พยาธิเข็มหมุด	20
2.	*Enterobius*	*Enterobius*	26

TCI (46 articles)

Excluded (27)

- Duplicates (n = 17)
- No full-text (n = 7)
- Not in Thailand (n = 2)
- Review (n = 1)

Included (n = 19)

- Duplicated with main databases (n = 3)
- Final included (n = 16)
