# Supplementary material for: Prevalence and Epidemiological Patterns of Enterobius vermicularis Infection in Thailand: A Systematic Review and Meta-Analysis
Source: Med Sci (Basel). 2025 Sep 24;13(4):207. doi: 10.3390/medsci13040207 (PMC12551123; doi:10.3390/medsci13040207)
Supplement: Supplementary file 1 [file medsci-13-00207-s001.zip › Table S4. Meta-regression and subgroup analysis_final.pdf]

Table S4. Meta-regression and subgroup analysis of the pooled prevalence of *E. vermicularis* infections in Thailand

1. Meta-regression analysis of the pooled prevalence of *E. vermicularis* infections in Thailand

Covariates	tau^2	Test for residual heterogeneity, *P* value	Residual heterogeneity I^2 (%)	Test of moderators, *P* value	Number of studies
Publication years	3.2734	< 0.0001	99.35	0.0037	56
Part of Thailand	2.2278	< 0.0001	99.06	0.0002	56
Age groups	2.3228	< 0.0001	99.15	< 0.0001	56
Male percentage	3.1558	< 0.0001	99.44	0.1595	46
Types of participants	1.8942	< 0.0001	98.85	< 0.0001	56
Method for *E. vermicularis* detection	0.6716	< 0.0001	97.14	< 0.0001	56

2. Subgroup analysis of the pooled prevalence of *E. vermicularis* infections in Thailand

Pooled prevalence	Subgroup	Test for subgroup difference	Pooled prevalence (%) [95% CI]	I^2 (%)	Number of studies
Overall			3.58 [2.14; 5.93]	97.7	56
Publication years		0.0470			
	2000–2009		4.75 [1.85; 11.63]	98.1	22
	2010–2019		4.33 [2.31; 7.99]	95.7	25
	2020–2023		1.15 [0.44; 2.95]	92.9	9
Parts of Thailand		< 0.0001			
	Central Thailand		7.93 [4.51; 13.57]	97.3	25
	Western Thailand		3.67 [0.28; 34.14]	97.9	2
	Northern Thailand		3.83 [0.76; 17.18]	97.8	8
	Northeastern Thailand		0.75 [0.29; 1.90]	91.0	10
	Southern Thailand		0.85 [0.19; 3.80]	84.2	4
	Eastern Thailand		3.40 [2.12; 5.40]	N/A	1
	Central, Western Thailand		7.39 [6.44; 8.47]	N/A	1
	Central, Northeastern Thailand		26.88 [22.82; 31.36]	N/A	1
	Central, Western, Eastern Thailand		21.91 [19.19; 24.89]	N/A	1

	Central, Northeastern, Northern, Eastern, Western Thailand		8.76 [6.56; 11.60]	N/A	1
	Central, Northeastern, Northern, Eastern, Western, Southern Thailand		0.52 [0.28; 0.97]	N/A	1
	All parts of Thailand		0.05 [0.02; 0.10]	N/A	1
Age groups of participants		< 0.0001			
	Children		7.05 [4.46; 10.97]	97.5	42
	Adults		0.45 [0.25; 0.82]	0.0	5
	Mixed age groups		0.40 [0.12; 1.32]	98.2	9
Participants		< 0.0001			
	School children		5.03 [2.86; 8.70]	97.8	34
	Children in communities		20.78 [19.04; 22.65]	5.5	2
	Hilltribe children		19.93 [4.85; 54.89]	95.4	2
	Villagers		0.27 [0.14; 0.51]	70.1	12
	Orphanages		11.40 [6.83; 18.42]	92.3	2
	Hilltribe children/Orphanage		17.73 [13.70; 22.63]	N/A	1
	Immigrant children		25.24 [21.72; 29.11]	N/A	1
	Karen students		15.49 [13.12; 18.20]	N/A	1
	School children/Orphanages		8.76 [6.56; 11.60]	N/A	1
Detection methods for *E. vermicularis*		< 0.0001			
	Scotch tape technique		12.88 [9.90; 16.59]	97.4	35
	Direct smear/Concentration method		0.33 [0.21; 0.50]	63.1	21

N/A, not assessed

3. Subgroup analysis of the pooled prevalence of *E. vermicularis* infections in several provinces of Thailand

**Regions of Thailand**	**Province**	**Prevalence estimate (95% CI)**	I^2 **(%)**	**Number of studies (subsets)**
Eastern Thailand		6.69 [2.52; 16.58]	94.6	2
	Chachoengsao	3.46 [0.49; 20.64]	95	3

	Chonburi	13.21 [8.77; 19.41]	N/A	1
Western Thailand		7.43 [1.83; 25.73]	94.7	5
	Kanchanaburi	4.52 [0.25; 47.26]	98.1	2
	Tak	6.94 [1.32; 29.30]	95.1	2
	Ratchaburi	22.22 [12.40; 36.59]	N/A	1
Central Thailand		9.12 [6.22; 13.18]	96.4	39
	Bangkok	16.50 [9.50; 27.14]	97.5	3
	Samut Prakan	13.94 [7.68; 23.98]	98.6	7
	Phitsanulok	6.64 [2.49; 16.52]	93.2	5
	Pathum Thani	10.05 [6.62; 14.97]	85.0	4
	Sukhothai	13.09 [3.23; 40.44]	96.3	3
	Samut Sakhon	5.08 [0.30; 48.91]	93.1	3
	Phetchabun	11.11 [7.73; 15.72]	N/A	1
	Nakhon Sawan	4.97 [2.50; 9.62]	N/A	1
	Uttaradit	4.76 [2.72; 8.20]	N/A	1
	Phichit	10.76 [7.93; 14.45]	N/A	1
	Khamphaeng Phet	3.69 [2.11; 6.39]	N/A	1
	Uthai Thani	3.06 [1.38; 6.64]	N/A	1
	Ang Thong	38.60 [26.94; 51.72]	N/A	1
	Nakhon Nayok	15.77 [13.85; 17.90]	N/A	1
	Nakhon Pathom	12.81 [9.57; 16.94]	N/A	1
	Bangkok, Pathum Thani	15.95 [12.97; 19.47]	N/A	1
	Phitsanulok, Uthai Thani, Kamphaeng Phet, Nakhon Sawan	19.98 [17.75; 22.41]	N/A	1
	Ang Thong, Ayutthaya, Suphanburi	0.29 [0.09; 0.89]	N/A	1
Northern Thailand		3.05 [0.60; 14.03]	97.4	9
	Nan	0.47 [0.13; 1.72]	63.6	3
	Chiang Mai	32.27 [17.99; 50.85]	98.3	3
	Mae Hong Son	7.25 [3.05; 16.26]	N/A	1
	Phayao	1.43 [0.20; 0.945]	N/A	1
	Lampang	0.00 [0.00; 1.0000]	N/A	1
Northeastern Thailand		0.90 [0.25; 3.16]	96.8	12
	Nakhon Phanom	0.68 [0.28; 1.63]	0.0	3
	Khon Kaen	0.13 [0.03; 0.51]	0.0	2

	Nakhon Ratchasima	1.80 [0.45; 6.95]	69.3	3
	Kalasin	0.49 [0.20; 1.17]	N/A	1
	Ubon Ratchathani	7.73 [5.49; 10.78]	N/A	1
	Buriram	52.08 [43.94; 60.12]	N/A	1
	Surin, Buriram	0.23 [0.06; 0.91]	N/A	1
Southern Thailand		0.85 [0.19; 3.80]	84.2	4
	Nakhon Si Thammarat	1.58 [0.19; 12.20]	87.6	2
	Satun	0.49 [0.07; 3.39]	N/A	1
	Krabi	0.45 [0.06; 3.14]	N/A	1
Central, Northeastern, Northern, Eastern, Western, Southern Thailand	Ang Thong, Nakhon Ratchasima, Khon Kaen, Nan, Chon Buri, Kanchanaburi and Phatthalung	0.52 [0.28; 0.97]	N/A	1
Central, Northeastern, Northern, Eastern, Western Thailand	Tak, Nan, Bangkok, Khon Kaen, Rayong	8.76 [6.56; 11.60]	N/A	1
All parts of Thailand	76 provinces	0.05 [0.02; 0.10]	N/A	1

N/A, not assessed.
