# Supplementary material for: Prevalence and Epidemiological Patterns of Enterobius vermicularis Infection in Thailand: A Systematic Review and Meta-Analysis
Source: Med Sci (Basel). 2025 Sep 24;13(4):207. doi: 10.3390/medsci13040207 (PMC12551123; doi:10.3390/medsci13040207)
Supplement: Supplementary file 1 [file medsci-13-00207-s001.zip › Table S5. Meta-regression and subgroup analysis_final.pdf]

Table S5. Meta-regression and subgroup analysis of the pooled odds ratio (OR) comparing the risk of *E. vermicularis* infection between male and female participants in Thailand

1. Meta-regression analysis of the pooled odds ratio (OR) comparing the risk of *E. vermicularis* infection between male and female participants in Thailand

**Covariates**	**tau^2**	**Test for residual heterogeneity, *P* value**	**Residual heterogeneity I^2 (%)**	**Test of moderators, *P* value**	**Number of studies**
Publication years	0.0160	0.0791	22.11	0.1745	29
Part of Thailand	0.0118	0.1821	19.57	0.0993	29
Age groups	0.0208	0.0512	28.54	N/A	29
Male percentage	0.0215	0.0655	28.10	0.1005	29
Types of participants	0.0243	0.0476	31.19	0.5457	29
Methods for *E. vermicularis* detection	0.0208	0.0512	28.54	N/A	29

N/A, not assessed

2. Subgroup analysis of the pooled odds ratio (OR) comparing the risk of *E. vermicularis* infection between male and female participants in Thailand

**Pooled prevalence**	**Subgroup**	**Test for subgroup difference**	**Pooled odds ratio (OR) [95% CI]**	**I^2 (%)**	**Number of studies**
Overall			1.03 [0.9; 1.14]	32.1	29
Publication years		0.1342			
	2000–2009		0.97 [0.82; 1.13]	40.3	11
	2010–2019		1.03 [0.90; 1.19]	6.6	14
	2020–2023		1.69 [0.99; 2.86]	22.1	4
Parts of Thailand		0.0507			
	Central Thailand		0.98 [0.87; 1.10]	25.9	14
	Western Thailand		0.88 [0.47; 1.64]	N/A	1
	Northern Thailand		1.35 [0.96; 1.89]	37.2	3
	Northeastern Thailand		1.24 [0.64; 2.41]	0.0	3
	Southern Thailand		1.71 [0.7; 4.05]	N/A	1
	Eastern Thailand		3.82 [1.23; 11.89]	N/A	1
	Central, Western Thailand		1.28 [0.95; 1.73]	N/A	1

	Central, Northeastern Thailand		0.69 [0.45; 1.07]	N/A	1
	Central, Northeastern, Northern, Eastern, Western Thailand		0.96 [0.51; 1.83]	N/A	1
Age groups of participants		N/A			
	Children		1.03 [0.92; 1.14]	32.1	29
Participants		0.8272			
	School children		1.01 [0.90; 1.14]	24.0	223
	Children in communities		1.17 [0.87; 1.58]	N/A	1
	Hilltribe children		0.85 [0.14; 5.11]	63.4	2
	Orphanages		0.68 [0.24; 1.93]	79.7	2
	School children/Orphanages		0.96 [0.51; 1.82]	N/A	1
Detection methods for *E. vermicularis*		N/A			
	Scotch tape technique		1.03 [0.92; 1.14]	32.1	29

N/A, not assessed
